# COMPOUNDING WEAKNESS IS ROBUSTLY ASSOCIATED WITH COGNITIVE FUNCTIONING

**DOI:** 10.1093/geroni/igad104.0365

**Published:** 2023-12-21

**Authors:** Ryan McGrath, Kelly Parker, Jeremy Hamm

**Affiliations:** North Dakota State University, Fargo, North Dakota, United States; North Dakota State University, Fargo, North Dakota, United States; North Dakota State University, Fargo, North Dakota, United States

## Abstract

Muscle strength has been linked to cognitive functioning, but the utility and discernment of absolute and normalized weakness cut-points for identifying persons at greater odds of poorer cognitive functioning has not yet been examined. We sought to determine whether being weak under multiple definitions was associated with greater odds of cognitive dysfunction. The analytic sample included 17,980 Americans ≥50-years (age: 64.8±10.0 years) from the 2006-2018 waves of the Health and Retirement Study. A handgrip dynamometer measured handgrip strength and the highest recorded measurement was included in the analyses. Sex-stratified absolute, body mass normalized, and body mass index normalized weakness cut-points were used. Individuals were categorized as having weakness under 0, 1, 2, or 3 cut-points. Persons with scores ≤11 on the Telephone Interview of Cognitive Status had any cognitive impairment, while those scoring ≤6 had a severe cognitive impairment. Separate covariate-adjusted multilevel logistic regression models were used for analyses. Persons classified as weak using 2 cut-points had 1.17 (95% confidence interval (CI): 1.02-1.35) greater odds for future any cognitive impairment. However, for those below all 3-weakness cut-points, the magnitude of the odds ratios elevated to 1.68 (CI: 1.44-1.94) and 1.74 (CI: 1.73-2.21) for future any and severe cognitive impairment, respectively. Being below more weakness cut-points increased the odds for cognitive dysfunction, which suggests combining definitions may better identify persons more at-risk for future cognitive impairment. Engaging in modifiable lifestyle behaviors that are conducive to preserving strength and maintaining a healthy body mass during aging may have implications for cognitive function.

